# Twist2 promotes ovarian cancer cell survival through activation of Akt

**DOI:** 10.3892/ol.2013.1316

**Published:** 2013-04-24

**Authors:** YUBIN MAO, JINFEI XU, GANG SONG, NINI ZHANG, HAO YIN

**Affiliations:** 1 Department of Pathophysiology in Basic Science, Medical College of Xiamen University, Xiamen, Fujian 361102, P.R. China; 2 Cancer Research Center, Medical College of Xiamen University, Xiamen, Fujian 361102, P.R. China

**Keywords:** Twist2, hypoxia-inducible factor-1α, ovarian cancer

## Abstract

Hypoxia-inducible factor-1 α (HIF-1α) is an important prognostic factor in ovarian carcinoma. Hypoxia contributes to tumor progression and is involved in the epithelial-mesenchymal transition (EMT). Twist2 is an EMT regulator, however, it remains poorly understood in ovarian carcinoma. The present study evaluated the expression of HIF-1α and Twist2 and further investigated whether Twist2 is involved in hypoxia-induced apoptosis in ovarian cancer. A series of matched paraffin-embedded tissue sections from human primary ovarian cancer and normal ovarian tissues were examined through immunohistochemical analysis, a Twist2-overexpressing stable ovarian cancer cell line was established and deferoxamine (DFO) was introduced to simulate hypoxic conditions. DFO-induced apoptosis was examined by fluorescence microscopy, MTT assays and flow cytometry. In addition, a western blot analysis was performed to examine the molecular mechanism(s) of action. Twist2 increased in epithelial ovarian cancers associated with HIF-1α expression. The acquired expression of Twist2 was able to promote the survival of ovarian cancer cells through Akt phosphorylation under DFO-induced hypoxic stress. The results suggest that Twist2 activates the PI-3K-Akt pathway to protect cells from apoptosis in a hypoxic environment. Moreover, Twist2 may be involved in the HIF-1α signaling pathway in ovarian cancer.

## Introduction

Generally, malignant tumor tissue is able to survive under harmful hypoxic conditions and may even acquire a more aggressive phenotype (1). An increasing amount of data shows that hypoxia-inducible factor-1α (HIF-1α) is an important biological marker in the evaluation of the prognosis of patients with ovarian carcinoma (2,3). HIF-1α overexpression induces tumor invasion and is associated with the repression of E-cadherin (4). In addition, HIF-1α is an important micro-environmental factor that induces the expression of certain epithelial mesenchymal transition (EMT) regulators, including Snail, Zeb1, SIP1 and Twist (Twist1), and coordinates the interactions among these EMT regulators (5). Hypoxia or the overexpression of HIF-1α induces EMT and metastatic phenotypes *in vitro* and *in vivo* (6,7).

Twist2, also known as Dermo1, is from the basic helix-loop-helix transcription factor family and has been demonstrated to be essential in mediating cancer metastasis (6). Twist2, which exhibits a >90% identical structure and function to Twist1 (8), is also known to facilitate the EMT in cancer (9). Twist2-driven EMT is critical in cancer progression and is able to markedly reduce E-cadherin expression (10,11). Twist2 is commonly overexpressed in ovarian cancer. In ovarian carcinoma cells, hypoxia induces the downregulation of E-cadherin via the upregulation of Snail (4). However, Twist2 remains to be investigated under hypoxia in ovarian cancer.

The upregulation of Twist2 is associated with HIF-1α expression in ovarian cancer. We further hypothesized that Twist2 assists in the survival of ovarian cancer cells under hypoxic conditions, as well as in inducing EMT. The present study investigated whether the exogenous overexpression of Twist2 promotes ovarian cancer cell survival under hypoxia. The results suggested that the Akt survival pathway was involved in the progression of deferoxamine (DFO)-induced apoptosis in ovarian cancer cells. Thus, Twist2 may activate the PI-3K-Akt pathway to protect cells from apoptosis under hypoxia.

## Materials and methods

### Materials

Mouse anti-Twist2 antibody was purchased from Abnova Biotechnology (Taipei, Taiwan). Mouse anti-Flag, anti-β-actin and anti-Akt antibodies and DFO were purchased from Sigma (St. Louis, MO, USA). Rabbit anti-phospho-Akt (Ser 473) antibody was provided by R&D Systems (Wiesbaden, Germany). Mouse anti-HIF-1α, anti-Bcl2, anti-Bad, anti-rabbit IgG and anti-mouse IgG antibodies were purchased from Santa Cruz Biotechnology (Santa Cruz, CA, USA). PI3-K inhibitor LY294002 was purchased from Calbiochem (San Diego, CA, USA) and the avidin-biotin complex (ABC) kits and the 3,3′-diaminobenzidine (DAB) substrate kit were purchased from Thermo Scientific (Waltham, MA, USA) and Pierce (Rockford, IL, USA), respectively. Formalin-fixed and paraffin-embedded normal ovarian tissues and ovarian carcinomas were selected randomly from the tissue bank in the Department of Pathology, Zhongshan Hospital, Medical College of Xiamen University, Xiamen, China. The research protocol and design were approved by the Ethics Committee of Xiamen University (ID no: 20091116). All clinical investigations were conducted according to the principles expressed in the Declaration of Helsinki.

### Immunohistochemical staining

Immunohistochemical staining was performed on regular paraffin-embedded sections. The samples were fixed in 10% buffered formalin and embedded in paraffin. The sections were then cut and immunohistochemical staining was performed as described previously (12). The 4-*μ*m thick sections were deparaffinized in xylene and rehydrated in graded alcohol and distilled water. Subsequent to antigen retrieval, endogenous peroxidase activity was blocked with 0.3% hydrogen peroxide in methanol for 30 min, followed by rehydration in phosphate-buffered saline (PBS) and incubation with 5% goat serum for 60 min to bind the nonspecific antigens. The sections were incubated overnight at 4°C with the primary antibodies. The immunosignals were detected using the ABC kit at room temperature. Subsequent to rinsing, the sections were incubated with DAB, counterstained with hematoxylin, dehydrated and mounted. To prepare the negative control, the primary antibody was replaced with normal mouse IgG. The sections were then analyzed through standard light microscopy. Positive cells exhibited brown granules in the cytoplasm or cell nucleus. The samples were scored based on the percentage of positive tumor cells and the staining intensity. The positive cell percentage was determined by calculating the percentage of positive tumor cells from the total observed cells; 0, <10% and 1, >10%. The intensity was determined by comparing the staining of the tumor cells; 0, no staining or ambiguous staining and 1, medium or marked staining. The two scores were multiplied to categorize the staining; 0, negative (−) and 1–2, positive (+).

### Cell culture and generation of Twist2-expressing stable ovarian cancer cells

The human ovarian cancer cell line HO-8910 was obtained from the Shanghai Cell Culture Collection (Shanghai, China). The cells were maintained in RPMI-1640 supplemented with 10% fetal bovine serum and penicillin/streptomycin. The full open reading frame of human Twist2 cDNA (Dermo1, NM_057179) was cloned into the pFlag-CMV2 mammalian expression vector (Invitrogen, Carlsbad, CA, USA), with the Flag tag in-frame at the N-terminal of Twist2. The Flag-Twist2-expressing plasmid and the pBabe-puromycin vector were co-transfected into HO-8910 ovarian cancer cells using the lipofectamine 2000™ transfection reagent (Invitrogen) according to the manufacturer’s instructions. The Twist2-expressing stable clones and the vector control clones were each obtained through selection with puromycin. The Twist2 expression levels in the selected stable clones were then verified through immunoblot analysis with Twist2 and Flag antibodies. The proliferation rate of the transfected cells was detected and compared with that of the vector control through viable cell counts, using trypan-blue staining.

### Cell morphological and viability assay

The cells were seeded in normal medium for 24 h and then incubated with serum-free medium, 100 *μ*M DFO and 0.1% (vol/vol) dimethyl sulfoxide (control), for 24 h. Next, the cells were fixed with 4% paraformaldehyde and incubated with 10 *μ*g/ml Hoechst 33258 (Sigma). Cell morphology was observed under a fluorescence microscope (Leica DMIRB, Solms, Germany). Cell viability was assessed via MTT assay as described previously (13). Assays were performed in triplicate for each group of cells under serum-depleted and DFO-treated conditions. Data are expressed as the mean ± SD. The cell survival rate (%) was calculated as follows: number of surviving cells in the experimental group / number of cells in the control group × 100.

### Detection of apoptosis via flow cytometry

Apoptosis was identified via flow cytometry analysis as previously described (13). Briefly, the cells were treated with 100 *μ*M DFO for 24 h. The DFO-treated and untreated cells were then harvested, washed twice with PBS and fixed in 70% ethanol at 4°C overnight. The cell pellets were suspended in propidium iodide staining solution (20 *μ*g/ml propidium iodide and 0.2 mg/ml RNase in PBS) and incubated for 30 min at 37°C. The samples were then analyzed through flow cytometry. Apoptosis was measured as the percentage of cells with a DNA content lower than that of the cells in the G_0_–G_1_ stage in the propidium iodide intensity-area histogram plot.

### Western blot analysis

Western blotting was performed as described previously (13). The Protein Assay kit for the protein quantity analysis was purchased from Bio-Rad (Hercules, CA, USA). The enhanced chemiluminescence detection system was purchased from Amersham (Arlington Heights, IL, USA). All antibodies are as described in the Materials and methods section.

### Statistical analysis

The results of the experimental studies are expressed as the mean ± SD. Statistical differences were analyzed by Student’s t-test using SPSS 10.0 software (SPSS, Inc., Chicago, IL, USA); P<0.05 was considered to indicate a statistically significant difference.

## Results

### Twist2 is co-expressed with HIF-1α in primary ovarian cancer

**Figure 1.**
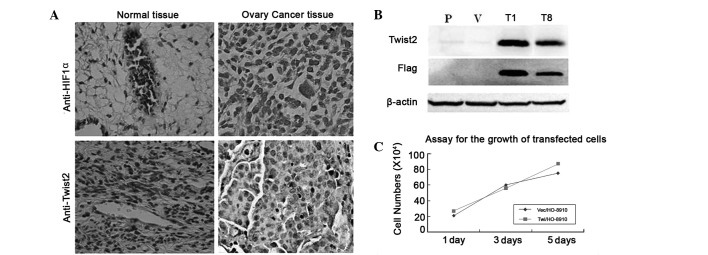
(A) HIF-1α and Twist2 were upregulated in ovarian cancer compared with normal ovarian tissues. Immunohistochemical staining for HIF-1α and Twist2. Specific staining was dark and nuclear counterstaining was light grey. Magnification, ×200. (B) Construction of Twist2 overexpression in the ovarian cancer HO-8910 cells. Two stable Twist2 overexpression clones (T1 and T8) and the vector group (V) and the parental group (P) were verified via western blot analysis with anti-Twist2 and anti-flag antibodies. (C) Proliferation characterization of Twist2-overexpressing HO-8910 clones and vector control clone. No clear changes were observed with the Twist2-overexpressing cells compared with the vector control in a growth assay. HIF-1α, hypoxia-inducible factor-1α.

A series of matched tissue sections from formalin-fixed and paraffin-embedded human primary ovarian cancer and normal ovarian tissues were examined via immunohistochemical analysis to assess the correlation between Twist2 and HIF-1α expression. The anti-Twist2 and anti-HIF-1α antibodies were shown to specifically recognize the corresponding proteins (Fig. 1A). Immunohistochemical staining was performed on 22 samples of primary ovarian cancer tissue and eight non-cancer ovarian tissue samples. The immunostaining analyses indicated the presence of high levels of Twist2 and HIF-1α in the areas containing the cancer cells of the primary ovarian tumors (Fig. 1). By contrast, Twist2 and HIF-1α were barely detectable in all the matched normal ovarian tissues. Overall, out of the 30 cases of ovarian tissue (including the primary ovarian cancer and normal ovarian tissues), 16 cases (53%) showed Twist2 positive expression and 18 cases (60%) showed HIF-1α-positive expression (Table I). Closer observation of the immunoreactivity for Twist2 and HIF-1α revealed the co-expression of Twist2 with HIF-1α in the tumor cells (r=0.451, P<0.05). These data demonstrated that Twist2 is commonly increased in ovarian cancers associated with HIF-1α expression.

### Twist2 overexpression in ovarian cancer exhibits survival advantages under hypoxia

The Twist2 expression in ovarian cancer patients was elevated compared with the control. The human ovarian cancer cell line HO-8910 was selected to further assess whether the stable overexpression of Twist2 in human ovarian cancer cells was able to alter cell survival *in vitro*. N-terminal Flag-tagged Twist2 and vector constructs were transfected into the HO-8910 cells. Following drug selection, two stable clones, Twi/HO-8910 (T1 and T8), and one vector transfected control, Vec/HO-8910 (V), were isolated and verified with specific antibodies against the Flag-tag and Twist2 (Fig. 1B). The overexpression of Twist2 in the HO-8910 cells showed no significant effect on proliferation compared with the vector control (Fig. 1C).

**Figure 2.**
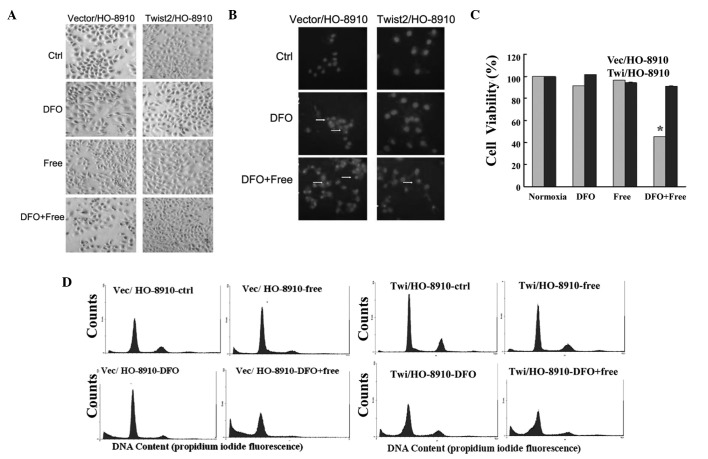
(A) Morphological changes in ovarian cancer HO-8910 cells under DFO-induced hypoxia. Cell morphology was observed 24 h after exposure to DFO. Compared with the untreated cells, the HO-8910 cells treated with DFO + free took on the typical appearance of apoptotic cells when observed under a phase-contrast microscope. The cells were observed and the cell micrographs were obtained in random microscopic fields (magnification, ×200). (B) Hoechst 33258-stained DFO-treated cells exhibited evidence of nuclear condensation and fragmentation through fluorescence microscopy (magnification, ×200). (C) Cell viability of the ovarian cancer HO-8910 cells induced by hypoxia. The HO-8910 cells were exposed to 100 *μ*M DFO for 24 h. The viable cells were quantified using MTT assays. Each column represents the mean ± SD; ^*^P<0.05 compared with normoxia control. (D) Demonstration of apoptosis in the HO-8910 cells through flow cytometry analysis following 24 h of DFO treatment. A lower sub-G_0_ rate was observed in the Twist2 overexpression group following treatment with the combination of DFO and serum starvation (Free). DFO, deferoxamine; Ctrl, control; Vec, vector; Twi, Twist2.

A hypoxic environment was then simulated using DFO. The Hochest33258-stained Vec/H0-8910 cells exhibited DNA condensation and nuclear fragmentation under 100 *μ*M DFO or the combination of DFO and serum starvation for 24 h. The Twi/HO-8910 group showed no clear morphological changes with DFO alone, but had fewer apoptotic cells once DFO was combined with the serum (Fig. 2A and B). In comparison with the control, Twist2 overexpression exhibited a higher level of cell viability under hypoxia combined with serum starvation, as measured with the MTT assay (P<0.05; Fig. 2C). A lower sub-G_0_ rate (27.16 vs. 42.10%) was demonstrated with Twist2 overexpression via flow cytometry (Fig. 2D). These data indicated that Twist2 had certain survival advantages under hypoxic conditions.

### Twist2 protects ovarian cancer cells from DFO-induced apoptosis through activation of the Akt survival pathway

**Figure 3.**
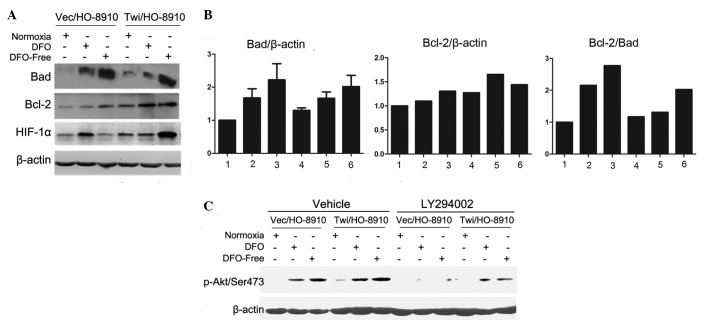
Twist2 is involved in DFO-induced apoptosis through the activation of the Akt survival pathway. (A) Expression of HIF-1α and the apoptosis-related Bcl-2 family with DFO treatment. (B) Relative expression of Bad to β-actin, Bcl-2 to β-actin and Bad to Bcl-2. Bars 1, 2 and 3, Vec/HO-8910 cells; bars 4, 5 and 6, Twi/HO-8910 cells; bars 1 and 4, control; bars 2 and 5, treated with 100 *μ*M DFO; Bars 3 and 6, combination of 100 *μ*M DFO and serum starvation. (C) Twist2 enhances resistance to apoptosis through the activation of Akt phosphorylation on Ser473. DFO, deferoxamine; HIF-1α, hypoxia-inducible factor-1α.

The changes in the Bcl-2 family proteins following DFO treatment were investigated (Fig. 3A), and the relative protein expression levels of Bad and Bcl-2 were analyzed using Image J analysis software (Fig. 3B). Once the ovarian cells had been treated with 100 *μ*M DFO or the combination of DFO and serum starvation, the expression levels of the pro-apoptosis protein Bad (relative to β-actin) in the Twi/HO-8910 (bars 5 and 6 vs. bar 4) and Vec/HO-8910 (bars 2 and 3 vs. bar 1) cells increased. Bad expression levels in the Twi/HO-8910 cells were lower than in the control group (bar 6 vs. bar 3, bar 5 vs. bar 2, bar 4 vs. bar 1). The expression levels of anti-apoptosis protein Bcl-2 (relative to β-actin) increased in the Twist2-producing group compared with the control group (bar 6 vs. bar 3, bar 5 vs. bar 2, bar 4 vs. bar 1). When the ratio of the pro-apoptosis protein, Bad, to the anti-apoptosis protein, Bcl-2, was compared, a clear decrease was observed in the relative expression in the Twi/HO-8910 group cells. This result indicated that Twist2 was able to protect the ovarian cancer HO-8910 cells from DFO-induced apoptosis by decreasing the Bad to Bcl-2 ratio.

The HIF-1α expression induced by the combination of DFO and serum starvation was much higher in the Twi/HO-8910 group. In addition, HIF-1α was more stable in the Twist2-producing cells under stress.

**Figure 4.**
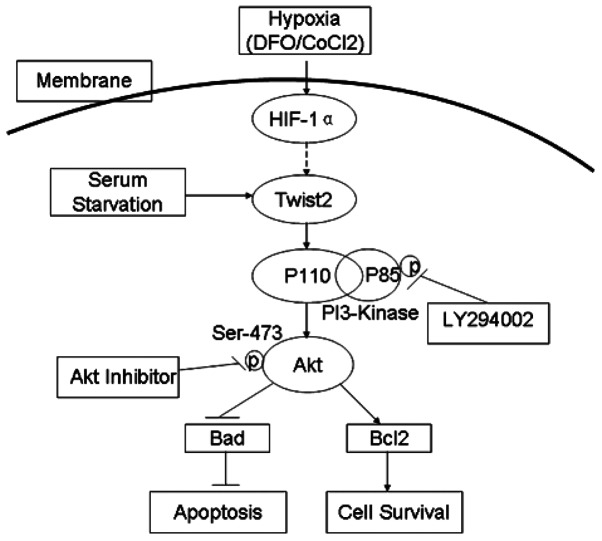
Proposed Twist2 involvment in ovarian cancer. When ovarian cancer cells are treated with DFO, HIF-1α is activated. Thus, Twist2 is directly activated in response to hypoxia or serum starvation. Twist2 then initiates the PI-3K-Akt pathway to assist in cell survival and anti-apoptosis under hypoxia. DFO, deferoxamine; HIF-1α, hypoxia-inducible factor-1α.

To further investigate these molecular mechanisms, the role of Twist2 in the activity of the PI3-K/Akt cellular survival pathway was examined by detecting the specific phosphorylation of Akt on Ser 473. As shown in Fig. 3C, the presence of Ser 473 phosphorylation indicated the activation of the Akt pathway, which was readily detected in the Twi/HO-8910 cells. The Ser 473 phosphorylation of Akt was downregulated in each group following pretreatment with LY294002 (PI-3K inhibitor). This result suggested that the Akt survival pathway was involved in the DFO-induced apoptosis of ovarian cancer cells and that Twist2 possibly activated the PI-3K-Akt pathway to assist in cell survival under hypoxic conditions (Fig. 4).

## Discussion

Tumor hypoxia is a common phenomenon in solid tumors (14). To survive in the stressful hypoxic environment, tumor cells develop a coordinated set of responses orchestrating their adaptation to hypoxia and even transform to the ‘metastatic’ phenotype through an EMT (6). HIF-1α is a critical mediator of the hypoxic response, which assists the hypoxic cells to compensate for hypoxia at the molecular level by increasing the activity of various host genes associated with apoptosis, erythropoiesis, angiogenesis and other survival pathways (2,15). HIF-1α is an independent adverse prognostic factor in ovarian cancer (2,16). Hypoxia contributes to tumor progression and is involved in the EMT by inactivating E-cadherin (7). Long-term hypoxia, which mimics the tumor microenvironment, drives a perpetual EMT through the upregulation of ZEB2, whereas short-term hypoxia induces a reversible EMT that requires the transcription factor Twist1 (17).

Twist2 is an EMT regulator (8,18,19). P12-induced EMT is mediated by Twist2 in hamster cheek pouch carcinoma (18). Previous studies have also shown that Twist1 and Twist2 cooperate with Ras or ErbB2 for complete EMT in breast epithelial cancer (9). In addition, the cyclin-dependent kinase inhibitor p21, which is involved in growth arrest, is directly regulated by Twist1 and Twist2 in the presence of E12 (20,21). Therefore, the expression of Twist2 was examined in the present study and observed to be upregulated in ovarian cancer (Table I, Fig. 1A). This observation is consistent with studies that showed that Twist2 is a potential diagnostic marker that promotes mesenchymal transition through the downregulation of E-cadherin in female patients with cervical carcinomas (11) and breast cancer (22). HIF-1α regulates the expression of Twist through the hypoxia response element (HRE) located in the Twist proximal promoter (6). Considering the similarity and overlap of functions between the two Twist proteins in development and cancer, we hypothesized that an association existed between Twist2 and HIF-1α. The present results showed that Twist2 was overexpressed along with HIF-1α in epithelial ovarian carcinomas (Table I, P<0.05, r=0.451; Fig. 1A). The same expression pattern of HIF-1α and Twist2 is also observed in tongue squamous cell carcinoma where it is associated with a shorter disease-free survival (23). Based on the correlation between the two molecules, we propose that Twist2 is involved in ovarian cancer hypoxia.

HIF-1α is the key cellular survival protein in hypoxic ovarian cancer (24). We hypothesized that Twist2 assists in the survival of ovarian cancer cells leading to a more aggressive phenotype under hypoxic conditions. Twist2-overexpressing stable ovarian cancer cell lines were then constructed. The results showed that Twist2 exhibited no effect on proliferation under normal culture conditions (Fig. 2C). The tumor microenvironment affects the progression and behavior of tumor cells. Thus, DFO was introduced to simulate hypoxic conditions.

In the present study, Twist2 was demonstrated to protect ovarian cancer cells from apoptosis under hypoxic conditions, as well as enhance cell survival in this unfavorable microenvironment. Twist2 promoted ovarian cancer cell survival when treated with DFO, particularly when combined with serum starvation. The results were demonstrated through flow cytometry (Fig. 2D). Subsequently, changes in the downstream Bcl-2 family proteins were investigated. The results showed that Twist2 was able to increase Bcl-2/β-actin expression and decrease Bad/β-actin expression in DFO-induced apoptosis (Fig. 3A and B). This result showed that Twist2 protected the cells from apoptosis by decreasing the Bad to Bcl-2 ratio

Numerous components of the PI3-K/Akt signaling pathway are involved in the regulation of HIF-1α. These components include PI3-K and PTEN. The activation of the Akt pathway is required to sustain cell survival traits under hypoxia (14,25). However, the exact role of PI3-K/Akt signaling in HIF-1α activation remains a matter of debate (24,26). Certain evidence suggests that the activation of Akt by hypoxia may depend on the cell type (27). Another study argues that PI3-K/Akt signaling is not involved in either the hypoxic or normoxic induction of HIF-1α (25). Our previous study showed that osteopontin was able to induce HIF-1α expression and promote HO-8910 cancer cell survival through Akt activation (28). Thus, the phosphorylation of Akt in cells expressing Twist2 and their corresponding parental cells was measured in the present study. The results showed that Akt was activated in the HO-8910 cells via Twist2 (Fig. 3C). In addition, the inhibition of the PI3-K/Akt pathway partly attenuated the Twist2-mediated Akt phosphorylation in the ovarian cancer cells. The present results are consistent with studies showing that Twist1 is able to directly upregulate the proto-oncogene AKT2 (8,29). Therefore, Twist2 may activate the PI-3K-Akt pathway to protect cells from apoptosis under hypoxia (Fig. 4). However, further studies should be conducted to investigate the detailed mechanism of Twist2 regulation by HIF-1α.

In conclusion, it was demonstrated that Twist2 was expressed at significantly higher levels in ovarian carcinoma cells and in correlation with HIF-1α. Twist2 promoted the survival of tumor cells through the PI-3K-Akt pathway, resulting in anti-apoptotic effects induced by tumor hypoxia. These results indicated that Twist2 is involved in HIF-1α signaling in ovarian cancer.

## Figures and Tables

**Table I.** Correlation analysis of Twist2 and HIF-1α expression in human ovarian cancer tissues.

	Twist2		
HIF-1α	(+)	(−)	Cases
(+)	15^a^	3	18
(−)	1	3	4
Cases	16	6	22

a Indicates the co-expressed cases of Twist2 and HIF-1α. χ^2^ =5.61, r=0.451, P<0.05. HIF-1α, hypoxia-inducible factor-1α.
